# Prevalence of Trachoma following Implementation of the SAFE Strategy in Three Local Government Areas of Taraba State, North Eastern Nigeria

**DOI:** 10.1080/09286586.2022.2045025

**Published:** 2022-03-30

**Authors:** Francisca Olamiju, Sunday Isiyaku, Nicholas Olobio, Hammed Mogaji, Ijeoma Achu, Nasiru Muhammad, Sarah Boyd, Ana Bakhtiari, Apake Ebenezer, Cristina Jimenez, Anthony W. Solomon, Emma M. Harding-Esch, Caleb D. Mpyet

**Affiliations:** a Mission To Save The Helpless (MITOSATH), Jos, Nigeria; b Sightsavers, Nigeria Country Office, Kaduna, Nigeria; c Federal Ministry of Health, Abuja, Nigeria; d Department of Animal and Environmental Biology, Federal University Oye-Ekiti, Ekiti, Nigeria; e Ophthalmology Department, Usmanu Danfodiyo University, Sokoto, Nigeria; f International Trachoma Initiative, Task Force for Global Health, Decatur, Georgia, USA; g Taraba State Ministry of Health, Jalingo, Nigeria; h Sightsavers, Haywards Heath, UK; i Department of Control of Neglected Tropical Diseases, World Health Organization, Geneva, Switzerland; j London Centre for Neglected Tropical Disease Research, London, UK; k Clinical Research Department, London School of Hygiene & Tropical Medicine, London, UK; l Department of Ophthalmology, College of Health Sciences, University of Jos, Jos, Nigeria

**Keywords:** Trachoma, trichiasis, prevalence survey, Nigeria, Taraba, Tropical Data, WASH

## Abstract

**Introduction:**

In 2019–2020, one round of antibiotic mass drug administration (MDA) was implemented for trachoma elimination purposes in Donga, Gashaka, and Ussa local government areas (LGAs) of Taraba State, Nigeria, following baseline surveys in 2009 (Donga and Gashaka) and 2013–2014 (Ussa). Here, trachoma prevalence post-MDA in these three LGAs is reported.

**Methods:**

In 2019 (Gashaka and Ussa) and 2020 (Donga), population-based, cross-sectional surveys were conducted following World Health Organization (WHO) guidance. A two-stage cluster sampling strategy was used. All residents of selected households aged ≥1 year were examined by Tropical Data-certified graders for trachomatous inflammation—follicular (TF) and trachomatous trichiasis (TT) using the WHO simplified trachoma grading scheme. Data on water, sanitation, and hygiene (WASH) access were also collected.

**Results:**

A total of 1,883 households participated. From these households, 4,885 children aged 1–9 years were enumerated, and 4,866 (99.6%) examined. There were 5,050 eligible adults (aged ≥15 years) enumerated in the same households, of whom 4,888 (96.8%) were examined. Age-adjusted TF prevalence in children aged 1–9 years was 0.22% (95% CI: 0.00–0.65) in Donga, 0.0% in Gashaka, and 0.19% (95% CI: 0.00–0.44) in Ussa. The age- and gender-adjusted TT prevalence unknown to the health system in adults aged ≥15 years was 0.08% (95% CI: 0.00–0.19) in Donga, 0.02% (95% CI: 0.00–0.06) in Gashaka, and 0.10% (95% CI: 0.01–0.18) in Ussa. In Donga, Gashaka, and Ussa, respectively, 66%, 49% and 63% of households had access to an improved drinking water source, and 68%, 56% and 29% had access to an improved latrine.

**Conclusion:**

In all LGAs, the elimination thresholds for TF and TT unknown to the health system have been attained in the target age groups. These LGAs should be re-surveyed after 2 years to show that reductions in TF prevalence have been sustained in the absence of MDA. Health authorities should continue to improve WASH facilities to reduce the risk of later recrudescence.

## Introduction

Trachoma is the leading infectious cause of blindness in sub-Saharan Africa and one of 20 neglected tropical diseases (NTDs).^1,2^ In 2020, there was an estimated global burden of 1.8 million cases of trachomatous trichiasis (TT).^3^ The disease is considered a public health problem in 44 countries of Africa, Central America, South America, Asia, Australia and the Middle East.^4^ Trachoma is caused by ocular infection with particular strains of the bacterium *Chlamydia trachomatis*. Transmission takes place when ocular or nasal discharges from an infected person are introduced to the eye of a susceptible contact, either directly person-to-person, via fomites, or aboard particular species of flies acting as mechanical vectors.^5–8^

In areas with intense transmission, infection is common in preschool-aged children.^9,10^ Infection is associated with a follicular and/or papillary inflammation of the upper tarsal conjunctiva known as active trachoma.^11^ Trachomatous inflammation—follicular (TF) and trachomatous inflammation—intense (TI) are specific signs of active trachoma, defined within the World Health Organization (WHO) simplified trachoma grading system.^12–14^ Repeated infections over many years may lead to recurrent chronic inflammation and the inside of the eyelid becoming scarred (trachomatous conjunctival scarring, TS). Over time, the scarred eyelid can turn inwards causing the upper eyelashes to rub against the eyeball (TT). This results in constant pain, light intolerance, scarring of the cornea and, when left untreated, visual impairment or blindness. Typically, the onset of visual impairment occurs late in life, but the age at which it begins probably depends on several factors including local transmission intensity.^15^

WHO endorses the SAFE strategy for trachoma elimination, which combines **S**urgery for TT, **A**ntibiotics to clear infection, **F**acial cleanliness and **E**nvironmental improvement to reduce transmission.^16^ Antibiotics are delivered via mass drug administration (MDA), with the number of annual rounds undertaken depending on the most recent TF prevalence estimate in 1–9-year-olds: one round when TF prevalence is 5.0–9.9%, three rounds when it is 10.0–29.9%, and five rounds when it is ≥30%.^17,18^ The decision to stop or continue implementation of MDA then relies on data from impact surveys, which WHO recommends should be conducted six to 12 months after the final planned round of MDA.^19^

To eliminate trachoma as a public health problem, three criteria must be met: (i) a prevalence of TT “unknown to the health system” of <0.2% in adults aged ≥15 years, in each formerly endemic district; (ii) a prevalence of TF in children aged 1–9 years of <5% sustained for at least 2 years in the absence of ongoing MDA, in each formerly endemic district; and (iii) evidence of a system to identify and manage incident TT.^20^

Nigeria, the most populous country in Africa, is trachoma-endemic.^21,22^ Baseline prevalence surveys conducted in Taraba state in 2009 and 2013–2014 estimated TF prevalence to be 6.1% in Donga, 5.0% in Gashaka, and 5.0% in Ussa local government areas (LGAs).^21,22^ Trachoma elimination activities, including community-based trichiasis surgeries, health education and one round of antibiotic MDA were commenced in these LGAs in 2019. Antibiotics offered were 1% tetracycline eye ointment for those aged <6 months or intolerant of macrolides, and oral azithromycin (Zithromax®, Pfizer, New York, NY, donated through the International Trachoma Initiative) for everyone else; estimated antibiotic coverage (determined by routine estimation during intervention delivery) was 82%, 80% and 82% in Donga, Gashaka and Ussa, respectively. MDA was undertaken between February and March 2019. In addition to MDA activities, F & E interventions were carried out across the three LGAs. Specifically, health educational messages focusing on usage of pit latrines in place of open defecation, installation and regular usage of tippy taps for facial cleanliness, regular handwashing after defecation and proper collection and disposal of refuse were rolled out during house-to-house visits. The activities were implemented by Mission to Save the Helpless (MITOSATH) and other stakeholders with support from The Queen Elizabeth Diamond Jubilee Trust, via Sightsavers. This paper reports the outcome of impact surveys conducted in these LGAs in November 2019.

## Methodology

### Study area

Taraba state, located in North-eastern Nigeria, has 16 administrative LGAs with Jalingo as the capital. The Onchocerciasis Control Unit, established in 1994, was subsequently expanded to become the NTD Control Department. It oversees the control activities targeted at major NTDs, including trachoma.

## Ethical considerations

The protocol was approved by the National Health Research Ethics Committee of Nigeria (NHREC/01/01/2007-09/8/2018C). Requisite approvals and field permits were obtained from the three LGAs. The London School of Hygiene & Tropical Medicine Ethics Committee provided approval (16105) for Tropical Data to support trachoma surveys. Standardized consent statements were read in local languages to the village head by trachoma survey teams upon their arrival to the community, to inform residents of the nature of the surveys and request permission to enter the community. Verbal informed consent was obtained from the head of the household and all participating adults prior to conducting interviews and examination, with assurance of strict confidentiality of survey data. Children aged <18 years provided verbal assent in addition to the verbal consent provided by their parents or legal guardians. Participation was completely voluntary, and no incentives were offered to prospective subjects. Consent to be examined was recorded in the Tropical Data app used for data collection on Android smartphones.

All individuals with active trachoma identified during survey work were provided with two tubes of 1% tetracycline eye ointment to apply twice daily for 6 weeks, and those with trichiasis were referred to the nearest health institution for surgical correction, and followed up.

## Field team training

Each field team included a trachoma grader and a data recorder, who were recruited by the local Ministry of Health and trained and certified according to Tropical Data protocols. Tropical Data (www.tropicaldata.org) is a global collaboration that supports health ministries to generate high-quality prevalence data to help with elimination efforts. Grader trainees were required to pass slide- and field-based inter grader agreement tests of diagnostic accuracy with a kappa score of ≥0.7, and recorder trainees were required to pass an electronic examination on the accuracy of their data capture with a pass mark of ≥90%.

## Study design, study population and sampling method

A population-based prevalence survey was conducted in each LGA, in accordance with WHO guidance.^23^ We projected that an enumerated sample of 1164 1–9-year-olds (after inflating the sample size by a factor of 1.2 to account for non-response) would be sufficient to estimate an expected TF prevalence of 4% with an absolute precision of 2%, assuming a design effect of 2.63.^23^ Fieldwork was carried out in November–December 2019 (Gashaka and Ussa) and December 2020 (Donga). Surveys in Donga were delayed due to security challenges that prevented field teams from conducting surveys as scheduled.

For each LGA, a two-stage cluster sampling method was employed. As an initial step, urban areas from both LGAs were excluded from the sampling frame as trachoma was not expected to be a public health problem in these settings.^17^ With the expectation of recruiting a mean of two children aged 1–9 years per household,^24^ a total of 25 first-stage clusters (villages) were selected using probability-proportional-to-village-size sampling.^17^ Each selected village was divided into compact segments^25,26^ of approximately 25 households. A ballot method was employed to select one segment, in which all 25 households were invited to participate. For the purposes of the survey, a household consisted not only of the basic family unit of parents and their children but was extended to include other adults and children living under the same roof and eating from the same pot.

## Data collection

In each selected household, after field teams explained the study procedures in detail, all residents aged ≥1 year were invited to participate. Consenting individuals were examined for trachoma using 2.5× binocular loupes, with illumination from the sun or penlight torch. Each eye was graded separately using the WHO simplified grading scheme, as amended following the 4^th^ Global Scientific Meeting for trachoma, November 2018, where the definition of TT was modified to exclude trichiasis that affects only the lower eyelid.^12–14^ Follicle size guides were used to aid TF diagnosis.^27^ In brief, a person with TT was defined as someone in whom, in at least one eye, one or more upper eyelid eyelashes touched the eyeball or there was evidence of recent removal of in-turned eyelashes from the upper eyelid.^12^ A person with TT unknown to the health system was defined as someone with TT but no previous offer of management of that condition by a health worker. A person with trichiasis was defined as someone in whom, in at least one eye, one or more eyelashes from the upper and/or lower eyelid touched the eyeball or there was evidence of recent removal of inturned eyelashes. Persons with trichiasis of the upper and/or lower eyelid were further examined for the presence of TS of the upper tarsus, and asked to provide information on whether they had been offered surgery or any other form of management for trichiasis in the past by any person employed by government to provide health care.^27^ Data on household-level water, sanitation and hygiene (WASH) access were collected through interviews and inspection (where relevant) of household sanitation facilities, using an adapted version of the WHO/United Nations Children’s Fund (UNICEF) Joint Monitoring Program (JMP) household questionnaire.^28,29^ WASH facilities were categorised as improved or unimproved, as per the WHO/UNICEF JMP definitions for monitoring progress towards the Sustainable Development Goals.^29^ All data were directly entered into the Tropical Data app on an Android smart-phone. Standard quality assurance and quality control measures for trachoma prevalence surveys^25^ were in place for all stages of survey implementation^30–32^

## Data analysis

Data analysis was performed using R (R Foundation for Statistical Computing, Vienna, Austria) and Structured Query Language was used for data cleaning. The 2006 Nigeria census data were used as the reference for data adjustment.^24^ The proportion of 1–9-year-olds with TF was adjusted for age at village level, in one-year age bands, while the proportion of adults with TT was adjusted for age and gender at village level, in 5-year age bands. The respective means of the adjusted village-level proportions provided the LGA-level prevalence of each sign.^28^ Confidence intervals (CIs) were determined by bootstrapping adjusted village-level proportions, with replacement, over 10,000 replications. Descriptive statistics in the form of percentages were used to characterise LGA-level access to WASH facilities.

## Results

### Enumeration and examination characteristics of study participants

In Donga, Gashaka and Ussa LGAs, respectively, a total of 628, 626 and 629 households participated in the study, with 1649, 1723 and 1513 children enumerated, and 1649 (100%), 1719 (100%) and 1498 (99%) children examined. There were 1548, 1735 and 1767 eligible adults (aged ≥15 years) enumerated and 1522 (98%), 1680 (97%) and 1686 (95%) examined from the same households Table 1. The number of children aged 1–9 years examined exceeded the target sample size in each LGA.

## Prevalence of TF and TT

**Figure 1:**
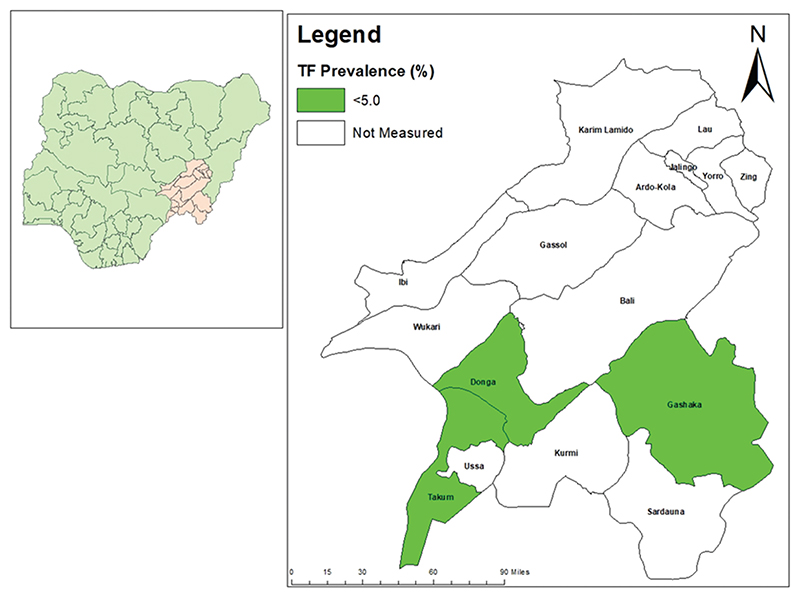
Prevalence of trachomatous inflammation—follicular (TF) in 1–9-year-olds, by Local Government Area, Taraba State, Nigeria, November 2019 (Gashaka and Ussa) and December 2020 (Donga). The boundaries and names shown, and the designations used on this map do not imply the expression of any opinion whatsoever on the part of the authors, or the institutions with which they are affiliated, concerning the legal status of any country, territory, city or area or of its authorities, or concerning the delimitation of its frontiers or boundaries.

**Figure 2:**
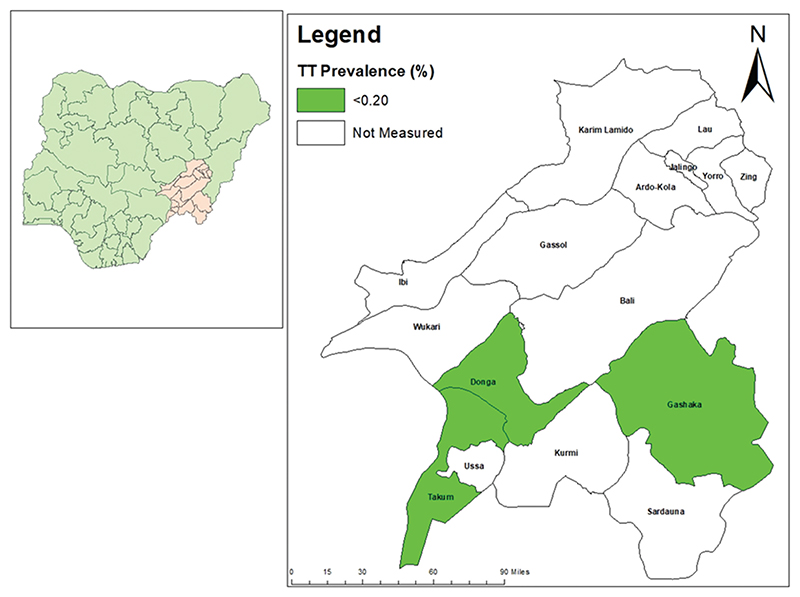
Prevalence of trachomatous trichiasis (TT) unknown to the health system in ≥15-year-olds, by Local Government Area, Taraba State, Nigeria, November 2019 (Gashaka and Ussa) and December 2020 (Donga). The boundaries and names shown and the designations used on this map do not imply the expression of any opinion whatsoever on the part of the authors, or the institutions with which they are affiliated, concerning the legal status of any country, territory, city or area or of its authorities, or concerning the delimitation of its frontiers or boundaries.

The age-adjusted TF prevalence in children aged 1–9 years in all LGAs surveyed was below elimination threshold Table 2 and Figure 1. In both Gashaka and Ussa, the age- and gender-adjusted prevalence of TT was equal to the age- and gender-adjusted prevalence of TT unknown to the health system for the same LGA. Age- and gender-adjusted prevalence of TT unknown to the health system in persons aged ≥15 years was below the elimination threshold for all three LGAs Table 3 and Figure 2.

## Access to water, sanitation, and hygiene (WASH) facilities

In Donga, Gashaka, and Ussa, respectively: improved drinking water sources were available to 66%, 49% and 63% of households; drinking water sources were within a 30-minute return journey of the house in 88%, 64% and 56% of households; and 68%, 56% and 29% of households had access to an improved latrine Table 4.

## Discussion

Periodically estimating the prevalence of trachoma in endemic areas is a necessary and important investment to determine when antibiotic MDA should be stopped.^31^ Baseline estimates of TF prevalence in these three LGAs justified one round of MDA.^17,18^ The present surveys provide evidence that TF prevalence is now below the 5% elimination threshold in each LGA. Following the guidance of WHO, pre-validation surveillance surveys should be planned to occur at least 2 years following impact surveys, in November 2021 (Gashaka and Ussa) and December 2022 (Donga) or later, to determine whether this sub-elimination-threshold TF prevalence is maintained in the absence of ongoing MDA.

The prevalence of TF estimated by these surveys is considerably lower than the corresponding baseline estimates (showing 96–100% decline).^21,22^ Though the 2009 baseline prevalence estimates in Donga and Gashaka were not adjusted for age, the apparent reductions following antibiotic MDA seen here might be compared to reported reductions in other settings, such as in Malawi, where TF prevalence fell 73–97% after a single round of MDA.^18^ When treatment was restricted to sub-village-level units in a cluster-randomised trial in the United Republic of Tanzania, one round of antibiotic MDA was less successful, with no significant difference in TF prevalence between the control and treatment group at either baseline (6.0 vs 4.3%) or after 12 months of follow-up (8.2 vs 9.3%), respectively. The Taraba programme achieved excellent therapeutic (>80%) MDA coverage in the three LGAs. This MDA was implemented several years after the baseline surveys, and it is therefore possible that prevalence might have fallen before MDA. It is also possible that high MDA coverage contributed to the difference between the TF prevalences in our surveys and those observed at baseline. As in other NTDs,^33^ based on first principles, WHO recommends high MDA coverage for achieving programmatically significant reductions in transmission of trachoma’s causative organism, though randomised trials to assess the impact of increased effort to maximise coverage of antibiotic MDA have largely been disappointing.^34,35^

It should be noted that although the data we report here provide circumstantial support for current practice in implementation of the SAFE strategy, they cannot be taken as evidence of effectiveness of those practices. The changes in TF prevalence from baseline to impact survey that we observed could reflect measurement error at either time point or a secular trend unrelated to the intervention.^36,37^ We note that in 2009, survey protocols, including training of the survey teams, were not as rigorous as in the Tropical Data system, so there may have been some measurement error in those surveys, as already alluded to. Genuine reductions in TF prevalence are associated with lower ocular *C. trachomatis* infection prevalence and infection load in children,^38^ which are accompanied by a decrease in community infection transmission intensity^39^ and, hopefully, by decreases in incidence of TT in the long run. The low TF prevalence estimates reported here are therefore good news for the Taraba state trachoma programme.

Facial cleanliness and environmental improvement are promoted as part of the SAFE strategy for eliminating trachoma as a public health problem. There is some evidence that access to improved latrines is associated with lower risk of trachoma, probably mediated through reduction in density of the muscid flies that serve as mechanical vectors for transmission of infection.^5,40^ In the Ussa survey, we recorded increased access to latrine facilities compared with baseline values.^22^ However, there were no baseline WASH survey data from Donga and Gashaka to allow further comparisons. One of the NTD road map 2021–2030 targets is 100% access to basic WASH services.^2^ Further WASH improvements are needed in all three LGAs: the majority of surveyed households did not have access to improved water and latrine facilities.

The prevalence of TT dropped from 0.8% at baseline to 0.10% at impact survey in Donga, from 6.5% to 0.02% in Gashaka, but remained unchanged at 0.1% in Ussa. Prevalence estimates of TT and TT unknown to the health system were below the elimination threshold at impact survey in all three LGAs surveyed; Donga (0.10% and 0.08%), Gashaka (0.02% and 0.02%) and Ussa (0.1% and 0.1%). These findings mirror those of Amnie *et al*.^41^ in Nassarawa State, and suggest that TT is not a disease of public health concern in Ussa LGA.^22^ However, in Donga and Gashaka, the apparent changes in TT prevalence over time could be due to the fact that prevalence at baseline was not adjusted for age and gender, overdiagnosis at baseline, or a secular trend unrelated to interventions.^36,37^ Reductions are likely to also have been partially driven by the trichiasis surgeries implemented in these areas.

Our data are encouraging from the trachoma perspective, but also highlight the remaining gaps in provision of water and sanitation facilities in this area. Furthermore, surveys in Donga were delayed due to security concerns, which pose a challenge for achieving elimination targets in Nigeria, as well as other countries contending with similar security issues.^42^ Achieving elimination status requires meeting set disease prevalence thresholds and sustaining them for at least a two-year period in the absence of further antibiotic MDA. During this period, a trachoma surveillance programme, including community and school screening for TT and TF, is planned.^41^ Since the prevalence targets of <5.0% TF in children aged 1–9 years and <0.2% TT in people aged ≥15 years have been met in these LGAs, efforts here should now be intensified to (1) transition structures to manage remaining and incident trichiasis cases; and (2) sustain low ocular *C. trachomatis* transmission rates, including mobilisation of resources to improve access to water and sanitation, with full community participation and ownership.^43^ These activities will improve the quality of life of the populace and sustain the gains in preparation for repeat population-based prevalence surveys in 2 years time.

## Figures and Tables

**Table 1:** Enumeration and examination characteristics of trachoma impact surveys in three local government areas (LGAs) of Taraba State, Nigeria, November 2019 (Gashaka and Ussa) and December 2020 (Donga).

				Persons enumerated, n			Persons examined, n (% of enumerated)	
LGA	Clusters	Households surveyed	Total	1–9 years	≥15 years	Total	1–9 years	≥15 years
Donga	25	628	3197	1649	1548	3171 (99)	1649 (100)	1522 (98)
Gashaka	25	626	3458	1723	1735	3399 (98)	1719 (100)	1680 (97)
Ussa	25	629	3280	1513	1767	3184 (97)	1498 (99)	1686 (95)

**Table 2:** Prevalence of trachomatous inflammation—follicular (TF) in three local government areas (LGAs) in Taraba State, Nigeria, November 2019 (Gashaka and Ussa) and December 2020 (Donga), with corresponding baseline TF prevalence data.^17,18^.

LGA	Baseline survey		Impact survey (November 2019 and December 2020)		
	TF prevalence in 1–9-year-olds (%)	Year of baseline survey	Children aged 1–9 years examined	Age-adjusted TF prevalence in 1–9-year-olds (%)	95% confidence interval
Donga	6.1	2009	1,649	0.22	0.00–0.65
Gashaka	5.0	2009	1,719	0.00	
Ussa	5.0	2013–14	1,498	0.19	0.00–0.44

**Table 3:** Prevalence of trachomatous trichiasis (TT) in three local government areas (LGAs) of Taraba State, Nigeria, November 2019 (Gashaka and Ussa) and December 2020 (Donga), with corresponding baseline TT prevalence data.^17,18^.

LGA	Baseline survey		Impact survey (November 2019 and December 2020)		
	TT prevalence in ≥15-year-olds (%)	Year of baseline survey	Individuals aged ≥15 years examined (n)	Age- and gender-adjusted prevalence of TT^a^ in ≥15-year-olds (%) (95% CI)	Age- and gender-adjusted prevalence of TT unknown to the health system^b^ in ≥15-year-olds (%) (95% CI)
Donga	0.8	2009	1522	0.10 (0.02–0.22)	0.08 (0.00–0.19)
Gashaka	6.5	2009	1680	0.02 (0.00–0.06)	0.02 (0.00–0.06)
Ussa	0.1	2013–14	1686	0.10 (0.01–0.18)	0.10 (0.01–0.18)

a Trachomatous trichiasis (TT) was defined as one or more upper eyelid eyelashes touching the eyeball or evidence of recent removal of in-turned eyelashes from the upper eyelid.

b TT unknown to the health system was defined as someone with TT but no previous offer of management of that condition.

**Table 4:** Household access to water, sanitation, and hygiene (WASH) facilities in three local government areas (LGAs) in Taraba State, Nigeria, November 2019 (Gashaka and Ussa) and December 2020 (Donga), with corresponding baseline data, where available.^15^.

LGA	Baseline survey (2013–2014)		Impact survey (November 2019 and December 2020)			
	Access to an improved water facility (%)	Access to an improved latrine (%)	Households surveyed	Number of households with an improved drinking water source (%)	Number of households with a drinking water source within 30 minutes return journey (%)	Number of households with an improved latrine (%)
Donga	Not reported	Not reported	628	414 (66)	555 (88)	429 (68)
Gashaka	Not reported	Not reported	626	307 (49)	401 (64)	350 (56)
Ussa	56	11	629	398 (63)	350 (56)	183 (29)
